# Can digital transformation reduce corporate stock price crashes?

**DOI:** 10.1371/journal.pone.0295793

**Published:** 2023-12-14

**Authors:** Xing Zhao, Xiangqian Li, Changman Ren

**Affiliations:** 1 School of Finance, Tianjin University of Finance and Economics, Tianjin, China; 2 School of Finance, Tianjin University of Finance and Economics, Pearl River College, Tianjin, China; 3 School of Business, Ningde Normal University, Ningde, China; The University of Hong Kong, HONG KONG

## Abstract

**Purpose:**

The purpose of this paper is to study the impact of enterprises’ digital transformation on the risk of stock price crashes, but also to study the mediating role of enterprises’ financialization and accounting conservatism in the enterprises’ digital transformation on stock price crash risk.

**Design/methodology/approach:**

Based on the data of 2,599 listed companies in China from 2010 to 2019, this paper constructs indicators of enterprise digital transformation through word frequency analysis method, and uses fixed-effects model and mediated-effects model to explore the impact and mechanism of enterprise digital transformation on the stock price crash risk.

**Findings:**

This study shows that firms’ digital transformation reduces the risk of stock price crashes and that financialization of firms and accounting conservatism play a significant mediating effect between enterprises’ digital transformation and the risk of stock price crashes.

**Originality/value:**

This study enriches the study of stock price crash risk by including digital transformation in the field of stock price crash research, and it examines the mediating roles of financialization of enterprises and accounting conservatism, which provides a new explanatory mechanism to the study of the correlation between digital transformation of enterprises and the risk of stock price crash.

## Introduction

A stock price collapse is a capital market anomaly with strong concealment, rapid contagion, and great destructiveness. It will not only affect market investor confidence and corporate interests but also undermine the stable development of the capital market. In addition, a stock price crash may also trigger systemic financial risks and even affect the real economy. Therefore, the risk of stock price crashes has become an important issue that has attracted much attention in the macroeconomic and microfinance fields in recent years [1].

Since Jin and Myers [2] proposed the theory of bad news hoarding that causes stock price crashes, scholars have conducted a great deal of research on stock price crashes, focusing on several dimensions, such as corporate management, the firm’s major shareholders and investors, the firm’s external regulation, informal institutions, Stock prices and dividends. First, for corporate management, corporate strategy [3–5], management regulation [6, 7], and corporate governance [8, 9] can affect stock price crashes. Secondly, major shareholders and investors, investor type [10] and institutional investor stability [11] affect stock price crashes. Again, in terms of firm external factors, audit fees [12], audit tenure [13], auditor industry specialization [14], and investor sentiment [15]. Finally, Political affiliation [16] and religious beliefs [17] inside the informal system can affect stock price crashes. In addition, there is a link between stock price [18], dividends [19, 20], and stock price crashes.

We can easily see from the preceding studies that scholars have undertaken many studies on the risk of stock price crashes and got good results. However, much past research has concentrated on individual corporate conduct and external regulation, with little attention paid to the impact of emerging technologies such as artificial intelligence, cloud computing, and digital transformation in stock market collapses. A few related papers only briefly talk about how digital transformation might cause stock prices to crash [21, 22]. They say that information and internal control are two possible mechanisms [23], they do not detail about the evidence and the channels of action between digital transformation and the risk of stock price crashes.

What can digital transformation bring to enterprises? Galindo-Martín et al. [24] pointed out that digital transformation enhances enterprise value creation, stimulates entrepreneurship, and brings digital dividends to enterprises. The network effects and business model innovations resulting from digital transformation can lower transaction costs, broaden marketing channels, and transform conventional business practices [25]. Hiding bad news from corporate insiders is a major cause of stock price crashes [2], and enterprises see digital transformation as a reliable solution. Digital transformation enables firms to collect, transmit, analyze, and apply data better than ever before, and automation and intelligence can enhance the comparability of financial information among firms, thus enabling outside investors to identify data anomalies in corporate disclosures and possible bad news concealment [26].

In addition to the immediate impact, there may be underlying mechanisms linking digital transformation with stock market crashes. A stock price crash occurs when stock prices suddenly and dramatically fall due to a concentrated disclosure of "bad news" [3]. This phenomenon not only diminishes investors’ wealth and makes them less inclined to make future investments, but it also affect the capital market’s healthy growth and enhances the total risk of the financial system. Corporate financial resources are a limited resource that can be produced and used over time. Financial assets can improve an enterprise’s balance sheet through increased investment income, enhancing its repayment ability and contributing to the enterprise’s market value [27]. The financialization of enterprises aids in easing financing restrictions, increasing capital utilization effectiveness and profitability, and reducing stock price volatility due to negative news hoarding [28]. In addition, Accounting conservatism also has the effect of reducing additional crashes. Accounting conservatism will accelerate management’s disclosure of bad news and delay the announcement of good news through asymmetric recognition of losses and gains. This behavioral consequence not only constrains management’s muffling behavior [29], but also reduces the degree of information force symmetry in the market and avoids the drastic effects that a concentrated outbreak of bad news can have on stock prices [30].

There is an underlying logical relationship between digital transformation, enterprise financialization, accounting conservatism, and stock price crashes, but past research has not shown the mechanism of their impact. Therefore, This study analyzes the influence of digital transformation on the likelihood of corporate stock price crashes in China’s A-share listed companies between 2010 and 2019. It using a fixed-effects model and a mediated-effects model to investigate the mechanisms of enterprise financialization and corporate accounting conservatism.

The expected research contributions of this paper are as follows: First, most of the existing studies on enterprise digital transformation focus on the economic consequences of enterprise performance growth, value enhancement, etc., while this paper extends enterprise digital transformation to enterprise stock market performance and integrates enterprise digital transformation and stock price crashes into a unified analytical framework, which enriches the related studies on enterprise digital transformation. Second, this paper uses the Python text analysis function and the word frequency analysis method to find the keywords related to enterprise digital transformation. It is conducive to scientifically and efficiently identifying the degree of enterprise digital transformation and improving the accuracy of the causality study in this paper. Third, this paper combines enterprise financialization and accounting conservatism as the microfoundations of structural problems in China’s current economic development process and conducts a comprehensive test of the two mechanisms of accounting conservatism and enterprise financialization, which provides a new perspective to deepen the understanding of the mechanism of the impact of enterprise digital transformation on the risk of stock price crashes and expands the research on the mediating effect of enterprise digital transformation on the risk of stock price crashes.

Part 2 then introduces the literature regression and presents the research hypotheses based on the relevant literature. The approach is described in Part 3 and includes sample selection, measurement of key variables, and model design. Part 4 contains the study’s findings, followed by a discussion. Finally, conclude the study’s findings.

## Literature review

### Digital transformation and stock price crash risk

As one of the core elements for the strategic transformation of enterprises, digital transformation is effective path for current enterprises to adapt to external uncertainties, actively participate in the integration of the digital economy and the real economy through digital technology, and reduce the redundant costs of traditional human resources and material resources, in order to promote the enterprise’s future high-quality development and sustained growth [31, 32]. Existing studies affirm the positive impacts of digital transformation, including enhancing technological innovation and business model innovation [33], improving innovation efficiency [34], boosting business performance [35] and improving social equity [36].

In addition to the repercussions described above, we must consider the impact of digital transformation on the collapse of corporate stock prices. This study aims to explore the following areas to address this issue.

On the one hand, In the process of digital transformation, enterprises disclose more information, which can minimize agency costs, increase internal control, and mitigate information asymmetry. Subversive reforms and changes in enterprise management and management systems will result from digital transformation, allowing enterprises to use digital technology to enrich the management system, effectively saving enterprise management costs, changing the relationship between internal floating and management, and reducing agency costs [37]. Digital transformation improves internal control systems, strengthens regulation, and reduces the scope for management to manipulate information, thereby requiring management to invest more effort and cost in information management. Chen et al. [38] show that the connectivity of the stock market shows how well the capital market shares information and helps with internal risk contagion and spillover effects. Therefore, digital transformation can replace inter-firm network connectivity and prevent intra-firm risk spillover. Wu et al. [39] provide similar evidence of the contagion function of global financial markets in terms of interconnectedness and crisis transmission. Moreover, artificial intelligence algorithms and recognition technologies can improve the quality of information by analyzing information about a company’s finances and operations in real-time and efficiently [40]. According to Wu et al. [41] digital transformation in enterprises enhances the efficiency of information processing and mining by optimizing the flow of information. Enterprises seek to enhance their external market support by promptly disseminating valuable internal information to external stakeholders. This practice facilitates a comprehensive understanding among market investors of the internal operations, production processes, sales performance, and other pertinent information [42]. Nambisan et al. [43] show that IT reduces the cost of information acquisition for external participation and mitigates internal and external information asymmetry.

On the other hand, Digital transformation enhances the level of risk-taking of a company as a way to reduce its operational risk. The company’s own business risk is one of the important reasons why the stock price crash occurs, and the higher the company’s business risk, the greater the volatility of its stock price [44]. According to agency theory, corporate management tends to be more risk-aversely motivated than shareholders. Managers with a risk aversion complex tend to avoid high-risk, high-return investment projects and take conservative decisions to reduce the firm’s current level of risk [45]. Whereas digital transformation reduces corporate agency conflicts through internal governance effects, thus increasing the level of corporate risk-taking [46]. And managers’ beliefs can influence firms’ cognitive decisions and outcomes in a crisis [47]. Thus, Risk-taking level is essentially an important characterization of the internal governance system of the enterprise, which means that the reshaping of the internal governance system of the enterprise has a tangible impact on the risk-taking level. With the advancement of digitalization, the enterprise management model, incentive mechanism, and good technology have supported the internal governance system. In addition, Yu and Huang [48] have found that uncertainty possesses a considerable degree of predictive power in determining anticipated stock returns. Hence, it is imperative for organizations to employ a systematic and accurate approach in balancing risk and return in order to optimize their risk-taking capabilities [49]. Throughout the digital transformation, businesses have integrated a new risk monitoring system to improve oversight of diverse business processes and management conduct. This measure has alleviated the management’s inclination toward excessive risk aversion [50].

### Digital transformation, enterprise financialization and stock price crash risk

The term financialization of firms is derived from financialization, which is the specific manifestation of financialization in microenterprises. Krippner sees financialization as the active participation of the real sector of the economy in financial investment activities [51], where the source of profitability is increasingly shifting from production and trade to financial investment.

To reduce the risk of a stock price crash, enterprises must always pay attention to a variety of potential risks, especially financial risk, high leverage, low financial condition, and other risks [52], through the optimal allocation of financial assets, improve the structure of capital operations, reduce the control of multiple risks mentioned above, and ultimately achieve the purpose of mitigating the sharp decline in stock prices. FinTech, as a brand-new industry at the intersection of traditional finance and digital technology, can meet the demand for resource expansion in the process of enterprise digital transformation. Fintech uses digital technology to absorb the market’s "many, small, and scattered" financial resources, considerably expanding the capital available for investment [53]. The study conducted by Bao and Haung [54] demonstrates the benefits of fintech lenders over conventional bank loans, particularly with regard to credit availability. At the same time, Fintech platforms provide multi-level and efficient capital flow channels and ways to reach enterprises, which may successfully address the traditional financial market’s supply financing problem [55].

Digital transformation refers to the changes brought by digital technology to the company’s business model, which in turn leads to changes in products, organizational structure, and process automation [56], while the core purpose of enterprise financialization is to improve the allocation of corporate financial resources and increase financial profit opportunities. With these two together, digital finance emerged. Digital finance is the effective combination of finance and Internet technology, relying on artificial intelligence, blockchain, cloud computing, big data, and other cutting-edge technologies with the advantages of "low-cost, high coverage, fast, and efficient" to maximize the credibility of the information in the stock price and improve the capital market’s information efficiency, thereby slowing down the drastic corporate share price fluctuations [57]. Digital finance in the provision of financial services has the characteristics of high efficiency and inclusion, and the alleviation of corporate financing constraints can help optimize the capital structure of enterprises, improve the corporate governance environment, and thus reduce the risk of stock price crashes [58]. Moreover, the development of digital finance has not only enriched the diversification of financing tools and services in the financial market but also greatly changed the pattern of market competition in the financial industry, which helps to provide better quality financial products and services and can effectively improve the accessibility of financial services [59]. The advancement of digital finance can alleviate corporations’ funding constraints and prevent them from purposefully concealing negative news, which can generate irregular changes in share values as they seek financing.

### Digital transformation, accounting conservatism and stock price crash risk

Accounting conservatism is a principle that addresses the recognition and measurement of accounting surpluses and losses. It is a significant indicator of the quality of financial reporting and an effective means of exerting governance impacts [60]. Basu defined accounting conservatism by building a model of surplus management [61]. He argued that good news needs to be supported by more evidence than bad news, and that this degree of asymmetry in profit and loss disclosure is accounting conservatism. Accounting conservatism can mitigate the negative impact of information asymmetry on the capital market, reduce the risk of stock price crashes, and limit the incentives and ability of managers to reduce the risk of exaggerating performance and hiding bad news from investors [62]. Moreover, conservative accounting policies can offset managers’ opportunistic behavior, reduce their incentives to manipulate earnings, and mitigate agency problems within the corporate [63]. The integration of digital technology into the production management process of enterprises can break the traditional boundaries of organizational structure, reshape the way of connecting internal and external enterprises, and realize the system interoperability and data interconnection of production and operation, equipment, products, resources, and decision-making systems. This helps to build an organizational framework for corporate governance based on smart contracts [64], thus creating a high-quality internal governance environment.

Accounting conservatism is a representation of corporate internal governance by improving corporate internal disclosure, which influences stock price collapse. Therefore, we analyze the following two points to explore the influential role of accounting conservatism:

First, The digital transition has resulted in increased corporate accounting disclosure and better caution. Data mining, storage, and analysis based on big data can rapidly transform standardized and unstructured information into standardized information, thus complementing traditional accounting disclosure [65]. Utilizing artificial intelligence algorithms and recognition technologies such as machine learning, we can analyze the massive multi-dimensional information on the company’s finances and operations in real time and with high efficiency, improving the quality of information [40]. Wu et al. [66] argued that the blockchain-IoT transaction model can automatically collect, upload, and record all relevant data in the process of enterprise transactions under the premise of satisfying certain assumptions, which improves the efficiency and quality of data collection and enhances the accuracy and timeliness of the recording of initial accounting information. Nie et al. [67] and other scholars found that enterprise digital transformation can significantly improve the comparability of enterprises’ accounting information by strengthening the quality of enterprise internal control, suppressing enterprise surplus management, and improving enterprise information asymmetry. Research by Zhao also shows the impact of business digitization on intelligent accounting information, arguing that digital technology using encryption algorithms can improve information security and transparency [68].

Second, Digital transformation undermines the hiding of bad news and makes corporate disclosures more truthful. Digital technology has increased the timeliness, transparency, and verifiability of data elements, which makes it more difficult for firms to mask unfavorable news through accounting manipulation practices [69]. Moreover, To edit or adjust the transaction information stored on the blockchain, the parties to the transactions on the chain must agree. For increased stability and reliability, all transactions and revenue recognition rely on predetermined circumstances and are dependent on blockchain data [70]. In addition, The application of digital technology has led to a more automated and intelligent way of recording, collecting and exchanging information between the internal and external parts of the enterprise, thus helping to improve the comparability of inter-company information and further identifying data anomalies due to the concealment of undesirable motives on the part of insiders [71]. Finally, Digital technology can also reduce the cost of information collection and processing for information users, prompting external watchdogs such as auditors, analysts, and institutional investors to identify corporate information manipulation, which in turn can deter management from hiding bad news [72].

By analyzing the contents of the literature review, we can anticipate that firms can use digital transformation to reduce agency costs, alleviate information asymmetry, and enhance risk-taking, thereby curbing the likelihood of stock price crashes. In this process, the financialization of firms and the Accounting conservatism play important roles in managing the risk of stock price crashes and serve as key mechanistic factors. Accordingly, this paper proposes the following research hypotheses:

H1: Enterprise Digital Transformation reduce Stock Price crash risk.

H2: In the path of digital transformation to reduce Stock price crash risk, enterprise financialization plays an intermediary role.

H3: In the path of digital transformation to reduce Stock price crash risk, Accounting conservatism plays an intermediary role.

## Methodology

### Sample

The data of Chinese A-share listed companies from 2010 to 2019 is utilized as the initial study sample in this work and processed as follows: (1) removing banks and financial companies; (2) deleting ST or *ST companies; (3) deleting companies with missing data; and finally selecting 10 annual observations from a total of 2,599 listed companies. The financial-related data of companies is from China Stock Market Accounting Research (CSMAR), and other data is from the WIND database (WIND). This paper winsorizes all continuous variables at the 1% and 99% quantiles to avoid the impact of extreme values on the results.

### Variable

Stock Price Crash Risk (DUVOL). Referring to existing studies [21, 23, 30, 73], this paper uses down-to-up volatility (Duvol) to measure the risk of a stock price crash, and the larger the value of Duvol, the higher the risk of a stock price crash for the company. After that, we replace Duvol with a negative coefficient of skewness (Ncskew) to test the robustness of the regression model.


$$NCSKEW_{i,t}=-\left[n{\left(n-1\right)}^{3/2}\sum w_{i,t}^{3}\right]/\left[\left(n-1\right)\left(n-3\right){\left(\sum w_{i,t}^{2}\right)}^{3/2}\right]$$ (1)

$$DUVOL_{i,t}=\log\left\{\left[\left(n_{u}-1\right)\sum w_{i,t}^{2}\right]/\left[\left(n_{d}-1\right)\sum w_{i,t}^{2}\right]\right\}$$ (2)

In the formula (1), n specifies the number of weeks in the year t when an individual stock i is traded. *n*_*u*_(*n*_*d*_) represents the number of weeks in year t when the particular weekly return of individual stock i is more (less) than the average specific weekly return for that year in the formula (2).

Digital Transformation (DT). In this paper, we use the analysis method of this paper to measure the level of digital transformation of enterprises [21, 22, 41]. Firstly, We organize the annual reports of all A-share listed companies on the Shanghai Stock Exchange and Shenzhen Stock Exchange through the Python crawler function, extract all the text content through the Java PDFbox library, and use it as a data pool to prepare for the subsequent feature word screening. Secondly, In terms of identifying the characteristic words of enterprise digital transformation, we drew on the research of Wu et al. [57] and further expanded the characteristic thesaurus of digital transformation concerning a series of classic literature, policy documents, and research reports on the topic of digital transformation, and finally formed five categories of characteristic words, including "artificial intelligence", "blockchain", "cloud computing", "big data", and "digital practice" and other five categories of feature words (Table 1). Finally, The corporate digital transformation index is calculated by using the Wingo database to count the frequency of words and then adding 1.

**Table 1:** Digital transformation keywords.

Cloud Computing Technology	Blockchain technology	Artificial Intelligence	Big Data Technology	Digital application technology
Cloud computing, streaming computing, graph computing, in-memory computing, secure multi-party computation, brain-like computation, green computing, cognitive computing, Fusion computing, Hundred million development, EB-level storage, Internet of Things, Cyber-Physical Systems	Blockchain, digital currency, distributed computing, differential privacy technologies, Intelligent financial contract	Artificial intelligence, business intelligence, image understanding, investment decision support systems, intelligent data analysis, machine learning, deep learning, intelligent robot, semantic search, Biometrics technology, face recognition, Automatic Speech Recognition, authentication, autonomous drive, natural language processing	Big Data, Data Mining, Text Mining, Data Visualisation, Heterogeneous Data, Credit reporting, Augmented Reality, Mixed Reality, Virtual Reality	Mobile Internet, Industrial Internet, Internet Healthcare, E-commerce, Mobile Payment, Third Party Payment, NEC Payment, Smart Energy, B2B, C2B, B2C, C2C, O2O, Netlink, Smart Wearing, Smart Agriculture, Smart Transportation, Smart Healthcare, Smart Customer Service, Smart Home, Smart Investment Advisory, Smart Culture and Tourism, Smart Environmental Protection, Smart Grid, Smart Marketing, Digital Marketing, Unmanned Retail, Internet Finance, Digital Finance, Fintech, Quantitative Finance, Open Banking

Accounting conservatism (CSCORE). This paper refers to Khan and Watts [74] accounting conservatism index to measure the level of accounting conservatism of Chinese listed companies. The specific process is as follows:

$$ESP_{i}/P_{i,t-1}=\beta_{1}+\beta_{2}D_{i}+\beta_{3}R_{i}+\beta_{4}D_{i}R_{i}+\varepsilon_{i,t}$$ (3)

$$GSCORE=\beta_{3}=\mu_{1}+\mu_{2}Size_{i}+\mu_{3}Mb_{i}+\mu_{4}Lev_{i}$$ (4)

$$CSCORE=\beta_{4}=\gamma_{1}+\gamma_{2}Size_{i}+\gamma_{3}Mb_{i}+\gamma_{4}Lev_{i}$$ (5)

Bringing (4) and (5) into (3) yields formula (6) below:

$$\begin{array}{c} ESP_{i}/P_{i,t-1}=\beta_{1}+\beta_{2}D_{i}+\left(\mu_{1}+\mu_{2}Size_{i}+\mu_{3}Mb_{i}+\mu_{4}Lev_{i}\right)R_{i}\\ +\left(\gamma_{1}+\gamma_{2}Size_{i}+\gamma_{3}Mb_{i}+\gamma_{4}Lev_{i}\right)D_{i}R_{i}+\varepsilon_{i,t} \end{array}$$ (6)

To get the accounting conservatism indicator for each company in a given year, we first do a regression on (6) across years and then plug the resulting regression coefficients into formula (5).

where ESP is earnings per stock; P is price per stock; R is the annual return on the stock; DR is a dummy variable that takes the value of 1 for the current year’s return R < 0 and 0 otherwise; Size is the natural logarithm of total assets; Mb is the book-to-market ratio; Lev is the Debt to Assets ratio; and i and t stand for the firm and year.

Financilization (FIN). Based on [76], this study calculates the extent of financialization of entity firms using the ratio of financial assets to total assets.

Control Variable. The control variables selected in this paper include firm size, Lev, RoA, firm age, Away, Sway, Soe, and Dual [23, 30, 73, 75, 76] (Table 2).

**Table 2:** Definition of control variables.

variable name	Variable Definition
**Size**	Total assets to natural logarithm
**Lev**	Total debts/total assets
**RoA**	Net profit/total assets
**Age**	Age of listing
**Awcy**	Average weekly-specific returns
**Swcy**	Standard deviation of week-specific returns
**Soe**	The company is a state-owned enterprise take 1, otherwise take 0
**Dual**	If the chairman and general manager are the same person, take 1, otherwise take 0.

### Model

To test hypothesis H1, this paper uses two-way fixed effects to construct the regression model (7). Two-way fixed effects models can account for unobservable characteristics at the individual level that do not change over time and may influence the probability of stock crashes. Individual qualities that do not change over time can be reflected by integrating fixed effects into the model, boosting the model’s explanatory power and accuracy.


$$DUVOL_{i,t}=\beta_{0}+\beta_{2}DT_{it}+\beta_{2}Control_{it}+\varepsilon_{i,t}$$ (7)

To test hypotheses H2 and H3, this paper adds Cscore and Fin to model (1) and constructs regression models (8), (9), (10), (11)

$$CSCORE_{i,t}=\beta_{0}+\beta_{1}DT_{i,t}+\beta_{2}Control_{i,t}+\varepsilon_{i,t}$$ (8)

$$DUVOL_{i,t}=\beta_{0}+\beta_{1}DT_{i,t}+\beta_{2}CSCORE_{i,t}+\beta_{3}Control_{i,t}+\varepsilon_{i,t}$$ (9)

$$FIN_{i,t}=\beta_{0}+\beta_{1}DT_{i,t}+\beta_{2}Control_{i,t}+\varepsilon_{i,t}$$ (10)

$$DUVOL_{i,t}=\beta_{0}+\beta_{1}DT_{i,t}+\beta_{2}FIN_{i,t}+\beta_{3}Control_{i,t}+\varepsilon_{i,t}$$ (11)

*i* = 1, …,*n* and *t* = 1, …,*t* for firms and years, and *ε* for the perturbation terms of the model.

## Empirical results

### Descriptive statistics

The descriptive statistics for the primary variables in this work are shown in Table 3. The mean values of DUVOL and NCSKEW are -0.171 and -0.263, respectively, with standard deviations of 0.468 and 0.698. This implies that there is a significant variance in stock price crashes among Chinese listed companies. The greatest and minimum values of enterprise digital transformation are 175 and 1, respectively, indicating that the degree of digital transformation varies between listed organizations. The average value of corporate digital transformation is 12.596, showing that most enterprises are not digitalized.

**Table 3:** Descriptive statistics.

Variable	Obs	Mean	Std. Dev.	Min	Max.
**DUVOL**	10978	-0.171	0.468	-1.286	0.986
**NCSKEW**	10978	-0.263	0.698	-2.27	1.566
**DT**	10978	12.596	28.489	1	175
**Cscore**	10978	0.012	0.029	-0.053	0.113
**FIN**	10978	0.032	0.062	0	0.353
**Size**	10978	22.127	1.169	19.94	25.722
**Lev**	10978	0.431	0.205	0.054	0.887
**RoA**	10978	0.039	0.062	-0.225	0.219
**Age**	10978	2.818	0.345	1.792	3.466
**Awcy**	10978	0.002	0.01	-0.016	0.034
**swcy**	10978	0.064	0.024	0.029	0.152
**Soe**	10978	0.316	0.465	0	1
**Dual**	10978	0.276	0.447	0	1

### Correlation analysis

Table 4 lists the Pearson correlation coefficients between variables, and the correlation coefficient between enterprise digital transformation and stock price crash risk is negative, which to some extent reflects that digital transformation mitigates stock price crash risk. Not only that, the correlation between stock price crash risk and accounting conservatism and enterprise financialization is negative, and all are significant at the 1% level of significance; the correlation between digital transformation and accounting conservatism and enterprise financialization is positive, and most of them are significant at the 1% level of significance, which preliminarily suggests that accounting conservatism and enterprise financialization are the possible path mechanisms for the digital transformation of enterprises to mitigate stock price crash risk. Finally, The results of the multicollinearity diagnosis indicate that there is no multicollinearity problem between the variables.

**Table 4:** Correlation matrix.

	DUVOL	DT	Cscore	FIN	Size	Lev	RoA	Age	Awcy	Swcy	Soe	Dual	VIF
**DUVOL**	1												
**DT**	-0.003	1											1.06
**Cscore**	-0.035***	0.006	1										3.39
**FIN**	-0.031***	0.069***	0.016	1									1.04
**Size**	-0.091***	0.011	0.766***	0.001	1								4.63
**Lev**	-0.073***	-0.104***	0.133***	-0.072***	0.528***	1							2.15
**RoA**	0.036***	0.002	0.143***	0.001	-0.007	-0.309***	1						1.24
**Age**	-0.067***	0.018**	0.02**	0.152***	0.176***	0.167***	-0.108***	1					1.12
**Awcy**	-0.145***	0.002	-0.067***	0.036***	-0.983***	-0.053***	0.156***	-0.059***	1				1.61
**Swcy**	-0.129***	0.091***	-0.198***	-0.004	-0.182***	-0.062***	-0.075***	-0.025***	0.575***	1			1.64
**Soe**	-0.077***	-0.124***	0.229***	-0.001***	0.308***	0.311***	-0.098***	0.173***	-0.051***	-0.093***	1		1.25
**Dual**	0.037***	0.082***	-0.108***	0.000***	-0.131***	-0.122***	0.028***	-0.089***	0.029***	0.048***	-0.267***	1	1.09

Note: *, **, *** represent significance at the 10%, 5%, and 1% levels, respectively.

### Baseline regression

The following fixed effects model regression results show that the regression coefficient of corporate digital transformation affecting stock price crash risk is -0.001, which is significant at the 1% level of significance, controlling for the rest of the variables. This implies the assumption that H1 of this study holds, i.e., enterprise digital transformation reduce stock price crash risk (Table 5).

**Table 5:** Baseline regression.

	DUVOL
**DT**	-0.001*** (-3.77)
**CONTROL**	YES
**COMPANY**	YES
**YEAR**	YES
**_cons**	1.363*** (3.43)
**F**	72.3
**Adj. R** ^**2**^	0.12

Note: *, **, *** represent significance at the 10%, 5%, and 1% levels, respectively.

### Robust test

In this paper, two methods are used for robustness testing. First, to ensure the reliability of the research findings, Ncskew is selected to re-measure the risk of stock price crashes for fixed effects regression concerning the existing literature. Table 6 shows that the regression coefficient of DT on Ncskew is -0.002, which is significant at the 1% level. This means that digital transformation reduces the risk of stock price crashes, which backs up the previous hypothesis H1. Second, We use digital transformation lagged by one period to re-measure the effect of digital transformation on the stock price crash risk, and the results show that the regression coefficient of LDT on DUVOL is -0.002, which is significant at the 1% level of significance. The results of the above robustness tests are all consistent with the previous results, which shows that the results of this study are robust.

**Table 6:** Robust test.

	NCSKEW	DUVOL
**DT**	-0.002*** (-3.61)	
**LDT**		-0.002*** (-3.83)
**CONTROL**	YES	YES
**COMPANY**	YES	YES
**YEAR**	YES	YES
**_cons**	1.727*** (2.90)	0.955* (1.66)
**F**	78.28	49.07
**Adj. R** ^**2**^	0.13	0.36

Note: *, **, *** represent significance at the 10%, 5%, and 1% levels, respectively.

### Endogenneity analysis

Although the above findings suggest that the digital transformation is linked to stock price crash risk, it is possible that the increased risk of stock price crashes for enterprises leads to the adoption of aggressive digital transformation strategies by enterprise managers in order to transform the enterprises’ negative image among investors, i.e., the digital transformation is a result of the stock price crash risk rather than the cause of it. This study explores two methods to alleviate the endogeneity problem and avoid this endogenous result.

First, We select the lag-one period of digital transformation as an instrumental variable and use the two-stage least squares method for endogeneity analysis. Table 7 demonstrates the regression results: the regression coefficient of DT on LDT is 0.667 and significant at the 1% level, in addition to the results of the Underidentification test and the Weak identification test, which both significantly reject the original hypothesis. From the coefficients in the third column, it can be seen that the regression coefficient of DT on DUVOL is -0.003, and the result is still stable at the 1% level.

**Table 7:** Endogeneity analysis.

	DT	DUVOL
**DT**		-0.003*** (-3.61)
**LDT**	0.667*** (60.63)	
**CONTROL**	YES	YES
**COMPANY**	YES	YES
**YEAR**	YES	YES
**_cons**	-90.655 (-6.70)	
**F**	490.82	51.60
**Adj. R** ^**2**^	0.88	0.07

Note: *, **, *** represent significance at the 10%, 5%, and 1% levels, respectively.

Second, This paper uses the propensity score matching method to deal with endogeneity. In this paper, the enterprise digital transformation mean is taken as the benchmark, and the samples are divided into (High DT) and (Low DT) groups and assigned the values of 1 and 0. After that, the nearest-neighbor matching method is used to find the treatment and control group [77]. After passing the balancing test, the matched samples were subjected to the regression test, and the results remained steady (Table 8).

**Table 8:** PSM result.

	Before PSM DUVOL	After PSM DUVOL
**DT**	-0.001*** (-3.77)	-0.002*** (-3.43)
**CONTROL**	YES	YES
**COMPANY**	YES	YES
**YEAR**	YES	YES
**_cons**	1.363*** (3.43)	0.482 (0.78)
**F**	72.3	27.03
**Adj. R** ^**2**^	0.12	0.11

Note: *, **, *** represent significance at the 10%, 5%, and 1% levels, respectively.

### Mechanism analysis

This paper explores the intermediate path from enterprise digital transformation to stock price crash risk from the perspectives of enterprise financialization and accounting conservatism. Specifically, we use Sobel Test mediation effect analysis to regress formula (8), (9), (10) and (11) to test the significance of the mediation effect. The Sobel test is a commonly used mediation effect test that can be employed to assess the impact of a mediating variable on the relationship between an independent variable and the dependent variable. In testing the impact of digital transformation on stock crash risk, by assessing the significance of the mediating effect, we can determine the mechanism role played by corporate financialization and accounting conservatism in the path of digital transformation affecting stock crash risk. This facilitates a more comprehensive comprehension of the causal relationship and the underlying mechanism of action between the variables. As shown in columns (2), (5) of Table 9, digital transformation contributes to enterprise financialization and accounting conservatism, and both are significant at the 1% significance level. And columns (3), (6) show that accounting conservatism reduces the risk of stock price crashes (β = -0.676, p<0.000), and enterprise financialization also has the ability to mitigate stock price crash risk (β = -0.138, p<0.000), i.e., accounting conservatism and enterprise financialization play a partially mediating role between digital transformation and stock price crash risk. Therefore, We can argue that digital transformation mitigates stock price crash risk by strengthening the level of enterprise financialization and increasing accounting conservatism.

**Table 9:** Mechanism analysis.

	(1) DUVOL	(2) CSCORE	(3) DUVOL		(4) DUVOL	(5) FIN	(6) DUVOL
**DT**	-0.001 *** (-3.77)	0.0001*** (6.70)	0.0001 (0.50)	DT	-0.001 *** (-3.77)	0.0001 *** (5.10)	0.0001 (0.78)
**CSCORE**			-0.676 *** (-4.24)	FIN			-0.138 *** (-1.94)
**CONTROL**	YES	YES	YES	CONTROL	YES	YES	YES
**COMPANY**	YES	YES	YES	COMPANY	YES	YES	YES
**YEAR**	YES	YES	YES	YEAR	YES	YES	YES
**_cons**	1.363*** (3.43)	-0.005 (-0.03)	22.75*** (6.21)	_cons	1.363*** (3.43)	-2.862 *** (-5.71)	16.982 *** (4.52)
**F**	72.3	158.30	45.90	F	72.3	45.53	44.51
**Adj.R** ^**2**^	0.12	0.12	0.04	Adj.R^2^	0.12	0.04	0.05

Note: *, **, *** represent significance at the 10%, 5%, and 1% levels, respectively.

### Heterogeneity analysis

According to the previous studies, digital transformation minimizes the risk of stock price crashes and has two channels of influence: enterprise financialization and accounting conservatism. In this part, we conduct group tests based on the level of enterprise external regulation to consistently validate our hypothesis.

First, Analysts are one of the self-constructed information transmission conduits between listed companies and investors, which has improved information asymmetry between listed companies and investors. Due to the relative advantages of specialized knowledge and processing of information collection, they can provide market participants with information that reasonably reflects the intrinsic value of the company, attenuates the price deviation in the market, reduces the degree of information asymmetry between the company and the investors, and lowers the cost of the contract, thus playing an important role in the capital market [78]. Analysts can utilize public market information as well as private information from company management, thus improving the accuracy of forecasts and corporate governance [79]. By revealing bad news hidden by company management and releasing it to the market in a timely manner, analysts help to alleviate information asymmetry and prevent the sudden release of bad news to the market after it has accumulated to a certain level, thus reducing the risk of a sharp fall in stock prices. digital transformation helps to improve analysts’ forecasting ability in terms of information sources and patterns [41, 65]. Therefore, we hypothesize that analysts’ forecasting will help reduce enterprises’ stock price crash risk. The results show that the regression coefficient for the analyst’s high prediction sample group is insignificant, while the regression coefficient for the low prediction sample group is -0.001, which is significant at the 1% level.

Second, Auditors, as integral actors in the realm of external regulation, fulfill their role in external governance through the provision of audit services of exceptional quality. Management is obligated to promptly provide pertinent information on the organization to effectively carry out its oversight duties and uphold the standards of corporate governance and information openness [80, 81]. By evaluating the reliability component of financial information, Li et al. [82] discovered a positive association between enterprises’ truthful excess management and the probability of future stock price crashes. According to another study, clients audited by organizations with sector knowledge have accounting surpluses that reflect "bad news" more quickly [83]. Table 10 demonstrates that the regression coefficients for the sample group of firms audited by the Big 4 are not significant, whereas the regression coefficients for the sample group of firms not audited by the Big 4 are significant at the 1% level, which is consistent with the analysts’ prediction.

**Table 10:** Heterogeneity analysis.

	High Analyst DUVOL	Low Analyst DUVOL	Big4 DUVOL	Non Big4 DUVOL
**DT**	-0.001 (-1.24)	-0.001*** (-3.01)	-0.001 (0.59)	-0.001*** (-3.54)
**CONTROL**	YES	YES	YES	YES
**COMPANY**	YES	YES	YES	YES
**YEAR**	YES	YES	YES	YES
**_cons**	-0.614 (-0.51)	1.752*** (3.69)	-4.215 (-0.94)	1.491*** (3.68)
**F**	9.45	36.35	1.77	56.88
**Adj. R** ^**2**^	0.09	0.12	0.13	0.12

Note: *, **, *** represent significance at the 10%, 5%, and 1% levels, respectively.

## Discussion

This paper looks at the risk of stock price crashes caused by a company’s digital transformation from the point of view of corporate information asymmetry. It also gives a new research perspective on the risk of stock price crashes caused by accounting conservatism and corporate financialization.

First, Table 1 in this paper confirms Hypothesis 1 by showing that enterprises’ digital transformation can significantly reduce stock price crash risk. Similar to our findings, digital transformation does have the ability to influence stock price crash risk [21–23]. The "information Hiding hypothesis" suggests that there is information asymmetry within and outside the firm and that management has the incentive to hide negative news and reduce the transparency of information about the firm, which is transmitted to the financial market in the form of a significant crash in the firm’s stock price [73]. Corporate information asymmetry is the main reason for the risk of stock price crashes [30, 84]. And the essence of digital transformation is to optimize the decision-making mechanism with the help of digital technology and reshape the information structure to promote intelligent, precise, and efficient enterprise management [85]. By applying digital technology to the process of storing and processing data and information, the digital transformation of enterprises improves the efficiency of information utilization and delivers enterprise-related information to market investors. This helps to alleviate the degree of information asymmetry between the internal and external parts of the enterprise and enhances the transparency and accuracy of information [41]. Furthermore, digital transformation enhances corporate information disclosure dynamics, which is conducive to reducing the degree of information uncertainty and asymmetry [86].

Since 2008, there have been numerous global stock price crashes, with 322 A-share companies in China experiencing a "flash crash" in 2021 alone. This not only harms investors’ interests but may also lead to stock price collapse through poor contagion, destabilizing the financial system, and inducing financial risks [87]. This study brings a decisive key to the solution of these problems, i.e., this paper sheds light on our understanding of corporate digital transformation to maintain the stability of the financial market by identifying the impact of enterprise digital transformation on stock price crashes. To reduce the space for negative news manipulation, improve internal control management, and reduce the risk of enterprise stock price crash, enterprises must strengthen the information disclosure mechanism of digital transformation and effectively disclose the information of digital transformation through regular financial reports, public announcements. Through digital transformation, enterprises can realize the improvement of their ability to collect, transmit, analyze and apply data, and thus better monitor and identify potential stock price crash risks. At the same time, digital transformation also helps companies establish a more standardized and transparent internal control system, reducing the possibility of insiders hiding bad news and lowering the risk of stock price collapse. Moreover, digital transformation is an important strategic choice. Managers can improve the competitiveness and stability of the entire economy by encouraging and supporting companies to undergo digital transformation.

Second, Table 9 confirms that the financialization of firms and accounting conservatism can play a mediating role in the risk of enterprises’ digital transformation and stock price crashes.

On the one hand, With the popularity and development of digital transformation, digitalization is more likely to enhance the depth and efficiency of financialization, which has a positive impact on the development of financial markets [88]. The rapid development of digital technology and the prosperity of the Internet have also made enterprise investment in financial assets more accessible, providing enterprises with a wider range of financial asset investment options [89]. Enterprise financialization reflects the phenomenon that enterprises rely more on the financial market for their investment, which is a manifestation of the enterprise’s preference for capital operations [90]. Firms allocate financial assets, especially more liquid ones, to hedge against uncertain future risks and sudden funding needs [91]. Firms have sufficient capital to avoid inefficient investment to a certain extent, which in turn affects the risk of a firm’s stock price crash. In contrast, the study by Sui and Yao [92] suggests that digital transformation inhibits the financialization of firms through link financing challenges. The probable reason for opposite results is the difference in the degree of digital transformation in enterprises. When digitalization is mature, with the use of the Internet of Things and cloud computing technology, investment institutions can use massive amounts of data to analyze and predict the business development trends of enterprises in real-time. while digitalization improves the degree of mismatch of enterprise credit resources, reduces the cost of enterprise financing, reduces the information cost of communicating with the outside world, and thus eases enterprise financing constraints.

On the other hand, Based on the information asymmetry theory, there is information asymmetry between information providers and users, which leads to information providers being able to interfere with the normal production and disclosure of accounting information by virtue of their information superiority [67], which in turn interferes with the quality of accounting information. With greater transparency and clarity in the disclosure mechanism, firms tend to report higher-quality accounting information [93]. Accounting conservatism is an important accounting information quality requirement, which is specified in accounting standards as "not overstating assets or earnings and not understating liabilities or expenses". Accounting conservatism can mitigate the negative impact of information asymmetry on the capital market [30]. However, Enterprise digital transformation can transform the books in each segment of the enterprise into structured and table-transformed information, which can not only provide data support for enterprise decision-making but also improve standardization and security in business processes and make enterprises more transparent in the implementation of accounting standards, which improves the quality of accounting information. Wu et al. [66] found that digital technology enhances the usefulness of accounting information by providing it in real-time and on-demand.

The process of economic globalization is increasing, and China’s financial market has been constantly updated and improved, but it is not yet mature and has a great deal of instability. This paper examines the extent to which an enterprise has adopted financialization and accounting conservatism. It finds that raising these levels can significantly enhance corporate disclosure, reduce information asymmetry and the agency problem, and prevent a stock market meltdown brought on by a wave of bad news. The deployment of digital transformation has improved asset configuration and liability management within firms, bolstered organizational financialization, and increased accounting conservatism The application of this plan not only reduces the firm’s financial risk, but also adds to the increase of its entire value, reducing the chance of a decrease in share prices. Digital transformation has improved firms’ ability to manage assets and liabilities effectively, maximize investment and financing options through data analysis and forecasting methodologies, and improve overall profitability and stability. In addition to providing new perspectives on how China’s capital market can reduce the likelihood of stock market meltdowns, this can also provide direction and support in maintaining stock market stability, avoiding financial risks, and encouraging China’s economic system to grow in a responsible manner.

Again, This study focuses on the role that external elements such as analysts and auditors have in digital transformation and stock price crashes, in addition to the internal corporate implications of digital transformation and stock price crashes. The findings presented in Table 10 demonstrate statistically significant regression coefficients for the subset of firms within the sample that possess poor analysts’ expectations and have not undergone auditing by the Big 4 auditors. These coefficients pertain to the relationship between the risk associated with digital transformation and the occurrence of stock price collapse. Analysts use specialized knowledge to make judgments about public and private information, to forecast company earnings, and to make recommendations to the market after issuing stock ratings, with the aim of conveying more information to the capital market, reducing the degree of information asymmetry, and improving the efficiency of the capital market’s operations [94]. Similarly, auditors have improved their ability to detect and curb management’s financial information behavior and, to some extent, the quality of the audit as they have gained experience in the industry [13, 14]. These outcomes reduce information asymmetry, boost the quality of accounting information, and lessen the likelihood of stock price crashes. This implies that analysts with higher expertise and professional audit firms possess an inherent ability to regulate stock price crashes, potentially mitigating the influence of digital transformation on such crashes. Conversely, analysts and audit firms with lower levels of professional competence exhibit reduced efficacy in governing stock price crashes, thereby amplifying the risk of stock price crashes resulting from digital transformation.

Finally, While the research in this paper is useful in giving governance to enterprise stock price crashes, we also need to be wary of the dark side of digital transformation [95, 96]. As pointed out [97], existing research related to tourism digitization mainly focuses on the benefits of digitization, while structural dependencies and digital control risks are often overlooked, which may lead to digital colonialism. The influence of digital transformation on the decline in stock prices of businesses is the main topic of this study. But digital transformation also highlights the hazards associated with digital dependency, including the possibility of digital system breakdowns and technological failures. Although this paper points out that the integration of internal and external information in enterprises through digital transformation reduces information asymmetry and reduces the risk of a stock price crash, the digital transformation of enterprises is a big project, and we should always pay attention to the digital risks that occur in the process. We can address the challenge of corporate digital dependency in future research by developing digital platforms and promoting corporate digital innovation, allowing digitalization to play a larger role in financial market research.

## Conclusion

Based on the theory of information asymmetry and internal corporate governance, this article investigates the impact of corporate digital transformation on stock price crash risk as well as the mechanism path of accounting conservatism and enterprise financialization. The results show that, firstly, Enterprise digital transformation can effectively suppress the risk of a stock price crash, and companies with higher levels of digital transformation have a lower risk of a stock price crash. Second, Accounting conservatism and financialization of enterprises mediate between enterprises’ digital transformation and the risk of stock price crashes, and the degree of influence is high. Finally, With respect to external regulatory heterogeneity such as analysts and auditors, the relationship between firms audited by Big 4 auditors and firms audited by no Big 4 auditors is significant in the relationship between firms’ digital transformation and stock crash risk for high predictive analysts but not for low predictive analysts.

The main theoretical significance of this paper is as follows: first, This paper constructs the degree of enterprise digital transformation through text analysis and word frequency research and links enterprise digital transformation with enterprise capital market performance, which enriches the research related to enterprise stock price crashes. Second, This study examines the causal pathway through which enterprise digital transformation affects the risk of stock price crash, focusing on the factors of enterprise financialization and accounting conservatism. By doing so, it offers a novel perspective to enhance our comprehension of the influence of enterprise digital transformation on stock price stability and broaden the scope of research in the field of digital transformation. Third, External regulators such as analysts and auditors play an important role in stock crash risk by protecting investors’ interests, providing important information, reducing risks, and promoting the effective operation of the market, thus helping to realize the healthy development of enterprises and also providing an important empirical basis for theoretical research in related fields.

Based on the above findings, this paper puts forward the following suggestions: first, Enterprises should seize the general trend of digital transformation, and in the environment of the digital economy, enterprises should accelerate the completion of digital transformation, improve internal control management, reduce the risk of stock price crash, and avoid the risk of enterprise operation. Secondly, Government departments should formulate and introduce guidance for digital transformation, set up typical digital transformation enterprises, provide relevant technical and financial support, encourage enterprises to accelerate digital transformation, and at the same time, strengthen the maintenance of the stability of the stock market in the process of digital transformation of enterprises, empowering the high-quality development of the real economy. Thirdly, The relevant departments should regulate and supervise enterprise accounting information and financialization behavior, improve the working mechanism for enterprise risk disposal, encourage enterprise stock prices to stabilize, and return major financial risks to a manageable range.

Finally, This paper examines the influence of digital transformation on the likelihood of stock price crashes. However, it is important to note that the securities market in China is still in the process of development, and there is a need for further enhancements in the relevant operational regulations and rules. Therefore, the generalizability of these findings to the United States, where the securities market is comparatively more advanced, requires additional investigation through future studies. Although the impact of digital transformation on the probability of stock market crashes is the main emphasis of this study, the stability of financial markets as a whole may also be significantly impacted by digital transformation. As a result, future research endeavors may seek to investigate the inherent correlation between the stability of financial markets and the process of digital transition. Additionally, these investigations may aim to elucidate the specific manifestations of this link within different nations and regions.

## Supporting information

S1 Data (XLS)
